# The Moral Force of Guidelines: How Surgeons Use Evidence‐Based Medicine to Curb Their Interventionism

**DOI:** 10.1111/1467-9566.70089

**Published:** 2025-09-30

**Authors:** Clay Davis

**Affiliations:** ^1^ Department of Sociology Northwestern University Evanston Illinois USA

## Abstract

The epistemology of evidence‐based medicine (EBM) is said to clash with the culture of surgery: EBM demands contemplation, whereas surgeons prize decisive, and even heroic, action. How, then, have surgeons come to embrace EBM? To answer this question, I analysed evidence‐based clinical practice guidelines (CPGs) and interviewed 15 attending surgeons at an academic medical centre in the United States. I find that surgeons use EBM to reconcile their interventionism with the changing demands made by their peers, patients, and payers in three moments that take place before, during, and after surgery, respectively. The first moment occurs at professional conferences where surgeons interpret ambiguous evidence in favour of action, embedding their instincts into EBM. The second moment unfolds in the clinic, where surgeons use EBM to communicate probabilistic risks to patients who expect surgeons to operate. The third moment transpires during recovery, when surgeons deploy EBM to shift the responsibility for action to paraprofessionals in line with payers' goal of shortening patients' hospitalizations. These findings challenge the notion that EBM merely standardises healthcare, rationalises patients' decisions or disciplines practitioners. Instead, I argue that evidence‐based CPGs are not only epistemic tools but moral ones that surgeons use to prompt, forestall and delegate action.

## 1 Introduction: Why Study Surgeons?

Evidence‐based medicine (EBM) has been a topic of interest for social scientists since the term became popular in the 1990s. Many early claims about the paradigm have been called into question in recent years: EBM does not merely threaten the autonomy of physicians (Denny 1999), who bend guidelines to their favour (Berg et al. 2000), embrace their uncertainty (shuster 2016) and use them to reproduce the physician's role as ‘sole decision‐maker’ (Timmermans and Mauck 2005, 20), nor does EBM equalises the treatment of patients, whom, instead, it racialises (Rondini and Kowalsky 2021), transforms into research subjects (Mykhalovskiy and Weir 2004), and rewards for ‘voic[ing] the loudest preferences’ (Rogers 2002, 102) or demonstrating their ‘risk acceptance’ (Ducey and Nikoo 2018). Further still, EBM has not standardized medical practice, which remains locally contingent and flexible (Hisham et al. 2016). This paper puts a finer point on the scholarly understanding of EBM by bringing to the fore a unique case: surgeons. I posit that by turning to surgeons, who have come to embrace EBM despite its clash with their professional culture (Pope 2003), one can better see that evidence‐based clinical practice guidelines (CPGs) are not only epistemic tools, but moral ones that help practitioners make fraught decisions.

The penetration of EBM into surgery has been the subject of little research beyond that which suggests surgeons clash with the rules‐based regime of EBM for at least two reasons: First, the performance of surgery is case specific, whereas EBM aspires to standardisation; surgeons see patients as individuals needing tailored solutions, whereas EBM prescribes uniform treatment to all patients fitting a given set of criteria. EBM cannot dictate the techniques employed in the operating room because the surgeons' decisions must reflect the ‘contingencies’ (Pope 2003) of both patients' preferences and their own skills. Second, there is an art to surgery that can only be inculcated to trainees tacitly; surgical education is based on the transfer of embodied skills that are not easily dictated (Schlich 2002; Prentice 2013). EBM is of limited use for training the next generation of surgeons, then, who must learn techniques by hand.

These two structural features of surgery culminate in a professional culture of interventionism. I use the term interventionism to describe surgeons' inclination to view patients' maladies as opportunities for curative action—their readiness to cut the flesh, identify the disease, and, ideally, excise or repair it. Interventionism is a heuristic that, as Katz (1999), argues, surgeons have used since the Napoleonic wars to act decisively under time constraints. Today, in an environment where most surgeons first meet their patients in outpatient clinics, surgeons' propensity to act is sustained not by the urgency of the battlefield but by the uncertainties, contingencies and demands of individual patients (Pope 2003) and the embodied skills that surgeons muster to address them (Schlich 2002). The resultant active posture is emblematic of the ‘surgical personality’ that, in Bosk's (1979) account, precludes the development of analytical dispositions, among them ‘expressions of doubt’ and ‘hesitation before action,’ that are integral to EBM.

Given the apparent incompatibility of surgeons' proactivity with EBM's deliberativeness, and my empirical focus within the U.S. healthcare system, one might expect evidence‐based medicine to have been foisted upon surgery from without by regulators or, perhaps, insurance companies. Yet, the number of professional organizations in surgery that have formed committees to draft CPGs suggests that surgeons have come to embrace the evidence‐based regime.

How do surgeons, then, reconcile their propensity towards action with the deliberations required by EBM? I find that at three empirical moments—before, during and after surgery—surgeons adapt evidence‐based CPGs to reproduce, mediate and reallocate their interventionist posture, respectively. First, in guideline committee meetings, surgeons exploit the ambiguity of uncertain evidence to embed their propensity towards action into EBM. Second, in the clinic, surgeons use evidence to quantify the risks of surgery and to communicate them to eager patients. Finally, after surgery, surgeons use CPGs to shift the responsibility for action to home care workers and patients themselves.

I will proceed with a two‐part review of relevant literature: I first evaluate the three dominant explanations for EBM's prominence in the sociology of health and illness and reject their applicability to surgery. I then turn to literature in the sociology of morality and use it to provide the basis for an understanding of EBM. I leverage Abend's (2014) concept of the ‘moral background’ to argue that evidence‐based guidelines are best understood as instruments that are not explicitly normative but that nevertheless shape how surgeons make moral determinations like when heroic surgery—that which poses a great risk but also a substantial reward—is justified.

## 2 Literature Review

### 2.1 The Limitations of Medical Sociology Literature on EBM

Medical sociologists have largely relied on three frameworks—standardisation, rationalisation and discipline—to explain the rise of EBM in fields of healthcare beyond surgery. The first explanation, standardisation, argues that medical research and its resultant CPGs harmonise clinical practice across space. This view traces EBM to the popularisation of the randomized controlled trial (RCT) in the mid‐20^th^ century United States by a vanguard of ‘therapeutic reformers’ who sought to enforce binding regulations around prescription drugs in order to bolster their professional status and autonomy (Marks 1997).

However, unlike therapeutic reformers, proponents of EBM in surgery have not, for the most part, tried to regulate their peers through top‐down organizations such as the Food and Drug Administration (Spodick 1975). Instead, EBM in surgery is decentralised: professional organizations issue CPGs which their constituent physicians elect to adopt. If this bottom‐up reform is standardisation at all, it resembles ‘regulatory objectivity’ which Cambrosio et al. (2006) describe as a set of conventions by which scientists mutually agree to be constrained. It appears at first glance that EBM is a case of regulatory objectivity: EBM requires an ‘unprecedented level of coordination,’ its guidelines, decision support tools, and checklists become ‘embedded regulations,’ and these CPGs become the substructure on which new knowledge is produced.

Yet, despite EBM, surgical practice remains unstandardised. Surgeons' choice of technique is highly dependent on local factors such as under whom one trained. For example, Cambrosio et al. themselves recognise that ‘In a field like surgery… the face‐to‐face transfer of operating skills plays a crucial regulatory role at the expense of more impersonal tools such as guidelines (Schlich 2002)’ (2006, p. 194).

A second way to think about EBM is that it removes substantive values—the nonscientific factors that creep into the exam room—from medical decisions. This rationalisation is simultaneously purported to reduce bias in medical decision making and criticised for ‘reduce[ing] patients to technological objects’ (Frankford 1994, 776).

However, EBM has not stripped values from medicine nor from surgery. In fact, evidence‐based guidelines are difficult to apply without them. At a clinic that treats intersex infants, Timmermans et al. (2019) find, for example, that physicians complement guidelines with ‘gender destinies’ that they ascribe to newborns. The flexibility of guidelines can also often work in favour of patients. Armstrong (2002) finds that physicians deferred to a rhetoric of patient‐centred medicine to resist the discipline of ‘decision support tools’ as they gained popularity in the UK.

The third explanation for the widespread adoption of EBM is that the paradigm disciplines doctors (and their patients) on behalf of insurance companies, hospital administrators, or regulators. In this reading, age thresholds for procedures such as mammograms and colonoscopies reduce the number of unnecessary tests that physicians order, checklists prevent negligence lawsuits and guidance on antibiotic use and sterile procedure reduces the number of (costly) hospital‐acquired infections.

However, this explanation does not adequately capture the role EBM plays in surgery. In the United States, surgeons maintain strong professional and practitioner autonomy and continue to enjoy the privilege of licencing their peers and restricting their ranks despite the decades‐long forward march of EBM (Darrow 2017, 183). In addition, although surgeons have paid for this professional autonomy by policing one another through disciplinary boards and, to a certain extent, CPGs themselves, these constraints are self‐enforced.

These three explanations for the rise of EBM overlap. Yet, even taken together, they cannot explain the steady ascent of EBM in surgery.

### 2.2 The Moral Dimension of Surgery

I posit, instead, that one must look to literature in the sociology of morality to understand why surgeons have come to accept EBM. EBM may not seem morally salient on its face, or one may only recognise its normativity to the extent that CGPs enforce standardisation, rationalisation and discipline. I argue, however, that EBM is moral in a more subtle and yet profound sense; it is part of the cultural scaffolding that supports moral reasoning that Abend (2014) calls the ‘moral background.’ In the moral background, EBM shapes second‐order moral issues such as how organizations constrain moral action (Jackall 2010), what categories of actors are considered worthy of moral consideration (Steensland 2010) and who has the power to set moral standards and the responsibility (Heimer and Staffen 1998) to meet them. The moral background into which EBM intervenes, of course, is already replete with moral precepts. Among them is surgeons’ propensity for action, which shifts surgeons' frame of action so that patients for whom the risks of undergoing the knife are substantial, but for whom the possible benefits of surgery are equally great, become candidates for surgery. The moral consequence of surgeons' tilt towards intervention has often been to lend hope to patients who have exhausted other medical options. EBM rearranges this corner of the moral background by giving surgeons new tools for making the decision of whether to operate, communicating with patients about the risks and benefits of surgery and allocating responsibility within and beyond the hospital. It is in the nuances of the moral background, where EBM interacts with surgeons' longstanding moral commitments, that I am best able to explain the paradigm's unlikely popularity in surgery.

I use Abend's notion of the moral background to extend earlier accounts of the second‐order morality of the hospital that have focused on its organizational dimensions. Bosk (1979) peers into surgeons' moral codes by describing how they classify errors. He finds that the most severe medical errors are those that stem from residents' disrespect of the hospital's hierarchical structure. Chambliss (1996), likewise, applies this organizational lens to nurses, whose moral lives, he argues, are constrained by hospital routines. Nurses' ‘powerlessness’ in the hospital bureaucracy may expose them to moral injury, but, Chambliss adds, it is also this passive role that allows nurses to distance themselves from their work by blunting their own emotional response when they fall short of their caretaking aspirations in resource‐limited settings.

I move beyond this organizational focus to study the role that epistemic instruments play in the moral background. To do this, I draw upon concepts from the interdisciplinary field of science and technology studies (STS) and work in the sociology of health and illness. In the first moment, at committee meetings and professional organizations, I reveal how surgeons, faced with the ‘interpretative flexibility’ (Pinch and Bijker 1984) of clinical research evidence, inadvertently circulate ‘academic urban legends’ (Rekdal 2014) that reaffirm their deference to interventionism. In the second moment, the clinical encounter, I describe how surgeons use evidence‐based guidelines to imagine new forms of ‘biosociality’ for their patients (Rabinow 1992). In doing so, I follow Mykhalovskiy and Weir's (2004) directive to look beyond EBM as precipitating the ‘erasure of the patient’ and to instead explore the ‘the role of the patient as a site for the production of evidence’ (1060). In the third moment, when EBM governs patients' recovery from surgery, I describe guidelines as ‘boundary objects’ that sit at the intersections between actors in the hospital and are, in turn, shaped by these actors' conflicts, alliances, and interactions (Star and Griesemer 1989). Together, these STS concepts allow me to recognise that evidence‐based guidelines are not only objects of knowledge but moral tools that surgeons use to imbue existing professional precepts with inertia, bring to the fore new ways of understanding risk and mediate interactions with payers.

## 3 Methods

The empirical basis of this study is 15 hour‐long interviews with attending surgeons—whose title indicates that they have completed residency training and who might be called ‘consultants’ in the United Kingdom—at a prestigious academic medical centre in the U.S. Midwest. I conducted interviews alongside my collaborator, Wayne Rivera, as part of a broader research project on surgeons' workplace challenges. Interviews were semi‐structured and, after probing for other sources of workplace friction, shifted to address three themes surrounding EBM: the production of evidence‐based guidelines, their clinical usefulness and their flaws. We recruited our sample across many specialities (called ‘sections’) within the surgery department.

The research protocol was given the status of ‘exempt review’ by Northwestern University's IRB on the condition that participants were given the option to forego having their interview recorded and to decline to be contacted for further research. Rivera and I deidentified respondents in our datasets and assigned them the pseudonyms that appear throughout this paper. We obtained access to the research site with the permission of an attending surgeon who also aided in recruitment but who was not privy to the list of participants we recruited or the data we collected.

The majority of the interviews were with colorectal surgeons, the section through which we initially gained access to the site. Colorectal surgeons were a useful sample for answering questions about EBM because, at the time of the study, the colorectal surgery section had partially adopted the potpourri of interventions that make up the ‘enhanced recovery after surgery’ (ERAS) protocol, the efficacy of which was a subject of internal division. In his study, Bosk (1979) used similar variation not to motivate a comparison, but to study the ‘underlying uniformity’ in surgeons' moral codes. I intend to make the same methodological intervention for the study of EBM by asking the following question: What components of ERAS do surgeons agree on and what does that consensus say about their shared moral background?

I analysed interview transcripts by identifying motifs of relevance to the sociology of health and organising them across the moments before, during and a surgery. I then adopted an abductive approach (Tavory and Timmermans 2014) to place these themes in conversation with one another, returning to the data iteratively to tease out threads that transcended them. I identified interventionism as the key overarching concept and used it to winnow which excerpts made it into the resultant paper. In studying evidence, I also adopted the STS method of epistemography (Dear 2001) which entails tracing the process of knowledge production (or, in this case, the drafting of CPGs) to study how epistemic artefacts become entangled in the social worlds of the actors from which they derive their facticity. In the final case of ERAS, I followed a CPG from the collection of its underlying data to its drafting and contested application.

I complemented interviews with a content analysis of CPGs published by 25 overlapping professional organizations (that represent the 13 subspecialities recognized by the American College of Surgeons) and their affiliated journals. In keeping with my focus on colorectal surgeons, I homed in on the CPGs for ERAS drafted by the American Society of Colon and Rectal Surgeons and the Society of American Gastrointestinal and Endoscopic Surgeons. I contextualised my interviews with surgeons about ERAS by analysing commentary pertaining to them in the ‘editorials,’ ‘viewpoint,’ ‘socioeconomic,’ ‘outcomes,’ and ‘reflections’ collections of the journal in which the guidelines were published, *Diseases of the Colon & Rectum.* My analysis of published material allowed me to extend my epistemography beyond the domains where knowledge is produced and into those where it is contested and its moral consequences laid bare.

### 3.1 First Moment: The Construction of Evidence‐Based Guidelines

In the following three sections, I report findings organised around the three moments before, during, and after surgery, respectively. In each, I choose illustrative CGPs and describe how they interact with surgeons' moral background as they are drafted, applied and revisited. I relay qualitative details from interviews that culminate in an image of EBM as part of a moral shift in surgery away from interventionism.

The first moment begins before evidence‐based guidelines are put into practice. The process of drafting CPGs, which unfolds outside of the hospital, is said to make surgery scientific. Yet, in surgeons' accounts of meetings at which guidelines were drafted, they recalled that the evidence that they were asked to evaluate was often ambiguous.

This was especially true in the early days of EBM when there were few if any randomized controlled trials (RCTs) of surgical interventions—ethical issues prevent the use untreated control groups in surgery—and second‐best epidemiological studies such as case control trials were not yet common in the field. In interviews, even surgeons with a favourable view of EBM commonly lamented the quality of older guidelines. One interviewee described a now discredited CPG that advised:

… patients who have [had] two episodes of diverticulitis should have surgery. The reason [was that] it was anticipated that with further episodes the colon would be prone to rupture and therefore you wanted to do the surgery before you have a catastrophic situation…

The rationale of this guideline was risk preemption; if a bad outcome was inevitable—in this case a ruptured colon—it appeared advisable to resolve it in advance. It was only by collecting his own experiential evidence that the attending, Dr. Klein, realized the guideline's premise was flawed:

[As] the years went on … I never got called from the emergency room that someone I'd seen with diverticulitis was now there with a rupture. …It seemed to me that everybody I was seeing who had [a ruptured colon] … [and who needed] emergency surgery … had never had [an episode of diverticulitis] before.

Klein became suspicious of the advice to preemptively operate and moved to investigate its evidentiary backing. He soon realized that the CPG was based on an ‘academic urban legend’ that had circulated widely after an initial false citation (Rekdal 2014):

… there was only one paper in all of the previous guidelines that had ever been cited … and it was from [Medical Journal] in 1968. And so I asked our library for it. They didn't have it. They had to … get an interlibrary loan, and the paper that was published had absolutely nothing to do with that issue. Someone once accidentally cited it and it got carried on … forever …

To correct this error, Klein conducted a chart review of nearly a thousand patients who presented at emergency rooms with diverticulitis. He found that, contrary to the bogus 1968 report, ‘almost all of the ruptures or the perforations [of the colon] are first episodes, so you cannot preempt [ruptures] if [they] happen the first time around.’ This result was a ‘game changer’ because it meant that surgery was indicated in far fewer cases.

Klein's correction was an about‐face from the prevailing guideline which, notably, reaffirmed surgeons' bias towards action. It appeared to Klein that the surgeons who had drafted the flawed guideline were unskeptical of evidence that reaffirmed their proclivity to act; they reproduced the interventionism that characterised their moral background in the resultant CPG. Klein's revision revealed the eagerness to act that characterised the surgical profession when the initial study was published in 1968 and the fact that this interventionism was initially sustained in part by EBM itself.

In another case relayed to me by an attending surgeon nearing retirement, Dr. Adams, the evidence that justified an interventionist CPG was not imagined, but rather misinterpreted. Adams described a guideline which stated that patients

should be kept above a certain [body] temperature. It was based upon evidence, but if you look at that evidence, that's not what [the authors] did and it's not the parameter that they looked at. They didn't say, ‘If you adjust the temperature to a certain level you achieve certain results.’ They said, ‘If you don't do anything and [the patient’s] temperature happens to be at this level, you get this result.’ It's a huge difference. And then to say, ‘Well, we're going to intervene and [raise the patient’s temperature] so that we can get that result’… The study wasn't truly designed for that.

This CPG, like that which Klein revised, suggests that its drafters, faced with the ‘interpretative flexibility’ (Pinch and Bijker 1984) of poor‐quality evidence, understood it in terms of their interventionist ‘surgical personality’ (Bosk 1979).

These early missteps in EBM reaffirm that guidelines can be understood as fixtures of the moral background. In the process of drafting and revising both CPGs, surgeons overcame epistemological ambiguity with moral conviction; they adapted imperfect evidence to fit their profession's values of preemption, intervention and hands‐on work. In doing so, these surgeons codified their moral commitment to action in new epistemic forms that remained durable until Klein and Adams revisited them decades later. Thus, an important lesson from the moment before surgery is that guidelines do not exert unilateral influence on surgeons by standardising treatments, disciplining practitioners or rationalising routines, rather, evidence‐based guidelines reflect, and ultimately crystalise, the values of the world in which they intervene.

### 3.2 Second Moment: To Cut or Not to Cut

If the first moment demonstrates that CPGs are made in the image of the moral background from which they stem; then, the second moment reveals that guidelines also go on to reshape that into which they intervene. After guidelines are drafted, surgeons must apply them in the clinic. The surgeons with whom I spoke unanimously agreed that evidence ought to play a role in their practice but differed in how it could be applied to their everyday decisions. A senior attending surgeon, Dr. Melnyk, explained this nuance well: ‘The evidence generated by our research is something that we should definitely use. The question only becomes *how* we should use it…?’

In subsequent interviews, following Melnyk's lead, I asked how respondents performed casuistry, the process of applying general rules like CPGs to specific cases, when it was demanded by EBM. For surgeons, my respondents agreed, casuistry or, as Melnyk put it, ‘apply[ing] [evidence] locally in the way that it makes sense,’ is difficult because it asks surgeons to ‘integrate’ their clinical expertise with abstract knowledge, a tension that has been at the heart of EBM since it was first debated by its earliest practitioners (Sackett et al. 1996). In interviews, it became clear that this reconciliation creates moral challenges, opportunities, and lenses. In particular, casuistry shapes the moral background by transforming *sui generis* encounters into moments when surgeons and patients alike are invited to compare themselves to their peers.

For surgeons, the form of moral comparison that EBM elicited was competition. In the moment of casuistry, EBM's guidelines yield vastly different results depending on which surgeon implements them. Klein used a sports metaphor to convey this point:

[When you] open up the newspaper it doesn't say, ‘What's the batting average of a 32‐ounce bat?’ You can't answer that question without saying, ‘Who's holding the bat?’ …In other words, we say, ‘Oh, it's evidence‐based medicine. You should do it robotically or laparoscopically.’ But…that's like saying ‘What's the batting average of a 32‐ounce bat?’ It's not really intelligible without knowing who's holding it.

As a result of this variation, EBM in surgery enforces an internal surveillance regime in which surgeons check their own success with a certain technique against that reported in the literature. Adams described his independent decision to use sutures rather than staples as based in a careful reading of published evidence:

I keep my own personal database on where I am and look at how it compares to what people are reporting in the literature … And right now, my leak rate is pretty good in comparison to what's written out there for any technique, right?

Adams's resistance to change reflects his membership in the group of colorectal surgeons that is resistant to EBM. However, seeing Adams's statement as an escape from EBM would be a mistake: Adams's decision to stitch rather than staple hinged on the comparison of his own performance to that of his peers—an attitude enabled by EBM. In his indirect adoption of EBM, Adams exemplifies part of what it means for guidelines to populate the moral background; their ubiquity has shifted how surgeons evaluate their own work even when they are not directly consulting CPGs. The decision to staple or to intervene at all is now routinised in a way that allows deliberative thought and comparison with colleagues.

EBM also asks surgeons to apply the same reflexive attitude to patients who, in the moment of casuistry, become echoes of the cases that preceded them. For patients, this perspective clashes with the fact that surgery is likely to be unfamiliar. The novelty of surgery means that, for some patients, going under the knife is a last resort and, for others, it appears to be the only permanent solution to their ailments even when surgeons prefer not to operate. The surgeons with whom I spoke noted that the latter patients were the most difficult to counsel; to these patients, a surgeon's disinclination to intervene would be seen as an abdication of their duty of care. I focus on these increasingly common cases in which surgeons prefer not to operate, and patients, conversely, demand it. This role reversal represents a departure from surgeons' interventionist cudgel and demands, instead, that surgeons immerse themselves in the moral background of EBM.

#### 3.2.1 Patients Who Worry

The most common reason that patients requested unwarranted procedures, in surgeons' telling, was anxiety. Melnyk recalled a patient whom he advised to forego surgery after his cancerous polyp was removed during a routine colonoscopy. Melnyk recounted the mild diagnosis:

The cancer was very small; it was in the polyp [phase], so the polyp was removed at the time of colonoscopy. … [B]ut there were some features that made the patient very concerned … And so he kept coming back and he was so worried that he still had cancer in his rectum that he wanted his rectum surgically removed and reconnected back to the colon. I advised him that that this could be a high‐risk surgery for a very, very low chance of having a cancer there.

In this anecdote, the stereotypical role of the surgeon and the patient appear reversed. The patient, in the surgeon's telling, expresses a zero‐tolerance policy regarding his polyp that resembles the ‘frames’ of ‘botheredness’ and ‘risk acceptance’ that Ducey and Nikoo (2018) found motivated patients' decisions to undergo elective surgery even in the era of EBM. The surgeon, conversely, sets aside his interventionism to instead advise caution; the risks of the procedure outweighed those of a possible cancer recurrence.

To take this tack, Melnyk turned to the moral affordances of EBM, invoking what he called ‘a Bayesian logic’ to explain that ‘the pre‐test probability [of finding cancer in the rectum] was extremely low.’ In Bayesian theory, the likelihood of an event, such as a cancer diagnosis, changes as new information about it becomes available. Melnyk's invocation of Bayes amounted to a moral manoeuvre because it lent him and his patient permission to rely on the limited information presently available. The Bayesian notion that ignorance is partial knowledge allowed Melnyk to embrace uncertainty, a state of affairs once seen as the enemy of the surgeon, and to forestall zealous action intended to gather new information through diagnostic procedures. Melnyk's casual invocation of a complex statistical concept also extends Timmermans and Angell's (2001) report that the ‘epidemiological’ logic of EBM introduces new uncertainty among resident physicians in the hospital. The more‐senior Melnyk uses Bayesian pre‐test probability, conversely, to set a quantitative threshold for uncertainty so that time‐sensitive decisions can be made without a bias towards action.

Yet, despite Melnyk's plea, the patient persisted; he wanted surgery. Melnyk recalled, ‘…we did his surgery, and it went really well. And because he … did not want to have a colostomy bag … about 10 days after surgery he already came back … [with] a large breakdown [where the rectum was reattached].’ However, it would be unfair to Melnyk, and dismissive of his patient's agency to say that the former's use of Bayesian probability failed to communicate the risks of proctectomy. A better conclusion would be that, regardless of the outcome, Melnyk's application of Bayesian probability to temper diagnostic uncertainty amounted to the use of EBM to tackle a moral challenge in surgery.

#### 3.2.2 New Forms of Belonging

The surgeons best able to consistently convince patients that surgery was not warranted drew not on EBM’s probabilism, however, but it's collectivism. They recognized that EBM provides patients with a sense of solidarity through the same mechanism that its critics argue it depersonalises them; EBM reminds patients that they are but one of many who have undergone this treatment in the past.

For EBM to serve as a source of belonging, I found, surgeons and patients alike had to be equally invested in CPGs and the evidence on which they are based. This buy‐in is most associated with guidelines that pertain to diagnoses such as breast cancer and that are the culmination of decades of struggle between patients, regulators and surgeons over what data count and whose experiences are valuable (Klawiter 2008). To that end, Dr. Miller, an attending breast surgical oncologist, was the surgeon I met who was the most enthusiastic about EBM. Miller told me that she routinely uses guidelines to correct patients' perceptions that radical mastectomies are necessary to preempt the return of cancer:

In breast [surgical oncology], the data is fantastic. … Breast cancer is like a decade or two ahead of a lot of other malignancies. I think we're actually doing our patients a disservice if we do not lean heavily on guidelines. … I often, in patient appointments, print off the NCCN [National Comprehensive Cancer Network] Guidelines and … I quote the literature to my patients. I explain to them how, you know, a lot of the movement in breast cancer has been towards doing smaller surgeries, which is in direct conflict with social expectations for breast cancer, surgery. Right? … Most controversy is around like doing bilateral mastectomies for one‐sided cancers. The data has never been good. But we've always just done it because we're like, ‘Oh, that's what she wants.’ But it's not actually making people live longer or live better. And we have data on that, too. So I lean really hard and say…’We have studies that show us this.’… I can't tell you what your lived experience will be, but I can tell you what thousands of women have done.

In reflecting on the current state of EBM, Miller, like Klein and Adams, looked back to the inertia of the moral heuristic of interventionism that preceded it. In the case of breast surgery, Miller acknowledged that it is difficult for patients and doctors alike to turn down a procedure such as a mastectomy after it has been codified as the standard of care in earlier CPGs. Miller's use of the term ‘expectation’ highlights how guidelines can also shape patients' moral background by informing their understandings of what they are owed by surgeons. In cases like these, updated guidelines need not only to ‘set the record straight,’ but to intervene in a moral background where decades of interventionism have led patients to see surgeons' action as a duty. Miller, when advising patients to forego radical mastectomy, distances herself from their expectations by reminding them that she ‘cannot tell [them] what [their] lived experience[s] will be.’ Instead, Miller empowers her patient to defer to what ‘thousands of women have done’ before her. With this rhetorical manoeuvre, Miller inducts her patient into a group that arises out of its members' shared biology, embodying what Rabinow (1992) calls ‘biosociality.’ Here, biosociality's power is in its transcendence not only of other categories of difference, but of space and time entirely, enjoining a patient with peers who lived before her and whom she will likely never encounter. Miller leverages this sense of solidarity to transform her patients' decision to forego surgery so that it reflects not the long‐held expectation of surgeons to intervene but an allegiance to a newfound community. Here again, casuistry's juxtaposition of the individual and group create moral opportunities for surgeons seeking to disrupt their profession's interventionism.

Another way that Miller uses guidelines to connect patients to each other is by reminding them of their role as a source of data that could improve treatment for those who will face the same illness in the future. Miller recalled that she often reminds patients that clinical data is important because it represents patients' contributions to progress in breast cancer treatment:

I always make the point of saying something to the effect of… ‘If I do not rely on the literature—if I do not quote you the data and inform your decisions—then I'm disrespecting all those people who enrol in trials.’ Right? If I don't take lessons learnt from those who went before us to make things better, we've disrespected their contribution…

Here again, Miller enrols her own patients in applying guidelines by reminding them of their duty to those who made sacrifices to produce the evidence on which EBM is based. In doing so, Miller imbues EBM with not only epistemic but moral authority. An implication of this finding is that surgeons who ask patients to participate in the creation of guidelines in some capacity may not be doing so only to earn ‘prestige,’ as Mykhalovskiy and Weir (2004) note, but also to give patients an opportunity to reciprocate the contributions of their peers and to further assimilate into their biosocial communities.

In both cases—Melnyk's counselling a patient to forego unnecessary surgery, and Miller's description of informing patients that the radical mastectomy is outmoded—guidelines emerge as more than merely a font of standardisation, rationalisation, or discipline. Although Miller's advice was heeded and Melnyk's was not, a direct comparison of their efficacy is unfair; a better conclusion is that EBM provides new ‘conceptual repertoires’ (Abend 2014) to the moral background of surgery that surgeons use to counsel patients about the risks and benefits of action. Melnyk drew on the abstract logic of Bayesian probability to dissuade his patient from using uncertainty as a justification for action, and Miller leveraged the collective nature of clinical research to reverse decades of ill‐founded interventionism in breast oncology. At an earlier time, both surgeons, lacking these vocabularies and heuristics, may have instead taken bold action to tame the unknown.

### 3.3 Third Moment: Recovery From Surgery

In the third moment, I combine my focuses in the prior two moments on the production and use of CPGs, respectively. I connect the drafters of a single CPG to those who apply it, revealing how moral assumptions that underlie the creation of CPGs become the basis of how surgeons reimagine their role in the hospital.

Although, in the popular imagination, surgery conjures gruelling images of the operating room, surgeons perform some of their most critical work during the postoperative period, when patients risk infection, pain, and other morbidities. In recent years, colorectal surgeons have sought to improve postoperative care by adopting a slew of evidence‐based guidelines called the enhanced recovery after surgery (ERAS) protocol. ERAS's drafters, the American Society of Colon and Rectal Surgeons (ASCRS) and the Society of American Gastrointestinal and Endoscopic Surgeons, intended the protocol to prevent minor complications and to reduce the total number of nights patients spend in the hospital. ERAS, by both measures, but especially the latter, has been effective: If a surgeon follows ERAS, a patient can expect to be discharged 3–10 days more quickly after their operation (Spanjersberg et al. 2011).

However, critics have cautioned that ERAS prioritises the wrong outcome. To these surgeons, ERAS may be ‘evidence‐based,’ but to measure the protocol's efficacy primarily on the narrow outcome of time to discharge is suspect. Adams, who was critical of EBM, questioned whether the programme's chosen outcome measure reflects ‘enhanced’ recovery from the perspective of the patient:

the wording enhanced recovery… is it that recovery is really enhanced? Or is it that the stay in the hospital shortened? Because that's really what you're talking about—length of stay, not really recovery. A different kind of thing from the perspective of the patient.

In asking not only whether ERAS improves outcomes but who determines what outcomes matter, Adams points to the moral background of ERAS. The sentiment that patients' experiences have been marginalised in clinical research is echoed by the editor of a volume on patient outcomes in *Diseases of the Colon and Rectum*:

…we have paid less attention to the most important data regarding surgical outcomes—those *provided* by our patients! … until recently, we did not ask the patient how our interventions affected their symptoms and their quality of life (Galandiuk 2022).

What actors, then, if not colorectal surgeons or patients, equate ‘enhanced’ recovery with a short hospital stay? ERAS’ emphasis on LOS can be attributed, as Cram (2019) argues of the measure's adoption more broadly, to ‘encouragement from payers and hospital administration’ who see freeing up hospital beds as a way to cut costs. The pressure on clinical researchers to measure efficiency spans hybrid and single payer healthcare systems. In the United States, clinical research published in *Diseases of Colon & Rectum* frequently reports LOS as a dual measure of both cost efficiency and patient recovery (Swenson et al. 2003). In the United Kingdom, researchers have justified their choice to measure ‘timely discharge’ by demonstrating, for example, that it aligns with the aims of the National Health Service's programme ‘Getting it Right the First Time’ (Han et al. 2022). In adopting short lengths of stay as the goal of ERAS, drafters of the protocol translated the moral goals of the market—the efficient allocation of scarce hospital beds—into the epistemological choice of outcome measure.

In time, payers' focus on LOS became embedded in the body of clinical evidence regarding ERAS at the expense of other measures of recovery. The authors of a Cochrane systematic review, on which the ERAS drafters relied, bemoaned that:

All studies [aggregated in our meta‐analysis] used length of hospital stay as the primary research question, implying this is a medically important parameter and reflects quality of recovery. No proof of this hypothesis exists[,] however…We feel [complications] should be the most important primary outcome parameter, since this is the only quantative measure of safety and the primary goal of an intervention‐comparison (Spanjersberg et al. 2011).

In relying on this limited literature, ERAS shifts not only who determines the goals of recovery from surgery but who is responsible for accomplishing them. In this dimension of the moral background that pertains to the allocation of labour, ERAS advances the broader trend of decentralising care work. Before the adoption of ERAS protocols, post‐operative care was managed by surgeons, their house staff or paraprofessionals within the walls of the hospital. Now, these tasks are assigned to patients themselves, who, as Klein reasoned, ‘[know] better [than I do] when they should be eating’ and to paraprofessionals who conduct home visits to prevent readmission (Stone et al. 2019). As a result, ERAS, like the gendered expectations that allocate responsibility for care after discharge from the neonatal ICU (Heimer and Staffen 1998), deepens a division in the moral background between the labour of care work and the science of surgical intervention.

Inside the hospital, ERAS has worked to reverse surgeons' interventionist posture. To prevent adverse events, colorectal surgeons have historically been proactive: they and their staff fed patients through nasogastric tubes, prescribed opioid analgesics, drained wounds, and cleared fluid from the bowels. However, these attempts by surgeons to hasten recovery or to control its trajectory sometimes prompted further intervention, creating a pernicious cycle of action. A resident whom Bosk (1979), 49) interviewed described how these interventions sometimes lead to further surgery and complications:

I couldn't help thinking that the phenobarbital I ordered for him postop may have given him the ileus that caused us to think he was obstructed. That made us reoperate, and then he gets this wound infection.

In our conversation about ERAS, Klein expressed wariness of these vicious cycles and lamented that surgeons need to recognise when their interventions are doing more harm than good. He explained:

… decisions in medicine largely break down to whether you believe your patients are getting better because of you or despite you. So, if you think they're getting better because of you, you do more stuff … If you think they get better despite you, you do less stuff.

Klein was among those in his department who advocated for ERAS. For Klein, ERAS represented no less than a new hands‐off paradigm in surgery. He articulated that while he ‘think[s] there's good evidence for enhanced recovery programs,’ he also ‘buy[s them] philosophically.’ The philosophy to which Klein refers, in his own words, and that ERAS reaffirms, is the notion that ‘the body's been around for millions of years’ and is, as a result, capable of healing itself. Most colorectal surgeons agreed; they were happy to scale back their duties if it meant hastening their patients' recovery. ERAS, then, served not only to codify the moral assumptions of payers and reallocate labour outside of the hospital but also to temper surgeons' longstanding notion that, all else being equal, action is better than inaction.

ERAS’ ability to effect this transformation was the result of a chain of events; payers' moral imperative to reduce patients' inpatient stays meshed with surgeons' willingness to let patients heal themselves. In the moment after surgery, then, CPGs act as ‘boundary objects’ (Star and Griesemer 1989) in that they were mutually shaped by, and shifted the moral boundaries (Lamont and Molnár 2002) between, surgeons, who conduct the research and apply it; nurses, who seek an expanding role in the hospital and beyond it and payers, such as Medicare, who set the outcomes of research through the back door.

## 4 Conclusion

I have argued that the advent of EBM has wrought not only organizational and epistemic transformations on the surgical profession in the United States but also changes to its ‘moral background’ (Abend 2014). In particular, EBM has reshaped surgeons' interventionism—a feature of the moral background that has historically slanted surgeons' clinical decisions towards action—by giving them new moral tools to cope with the challenges of the changing hospital.

EBM interacts with surgeons' interventionism differently across the three moments before, during and after surgery: While drafting early guidelines, surgeons exploited ambiguities in research evidence to codify their propensity to act in CPGs. The resultant CPGs, like the one that instructed surgeons to preemptively remove the sigmoid colons of patients with diverticulitis, persisted as the epistemic scaffolding of surgeons' moral background until, decades later, surgeons such as Klein conducted research that supplanted them. In clinical encounters, surgeons used the casuistry demanded by EBM as an opportunity to connect their patients to those who have experienced similar circumstances in the past. The seemingly neutral epistemic features of EBM—such as Bayesian probability—became moral tools that helped surgeons contextualise the risks and benefits of action and place patients in communities of their peers. In addition, after surgery, surgeons set the goals of evidence‐based guidelines in a way that enrolled new actors to aid in patients' convalescence. CPGs such as ERAS acted as intermediaries that aligned the moral values of payers, surgeons, and patients. In these three moments, CPGs contributed new conceptual repertoires to the moral background where they interacted with their pre‐existing counterparts like interventionism.

For sociologists of health and illness, the broader implication of this paper is that attention to the moral background permits one to see beyond the hypotheses that EBM has standardized, rationalised, or disciplined healthcare. I do not mean to dismiss EBM's role in these transformations, but instead to suggest that studying the moral backgrounds of medicine may help explain the wide variation in their consequences. Those who have studied the rationalisation of medical care, for example, have found that, in some cases, EBM depersonalises patients, whereas, in others, adherence to guidelines prevents biases from jeopardising their care. The divergence of these two findings may be explained by the fact that rationalisation initiatives introduce moral heuristics, tools, and frames that are more apt in some domains than others. I encourage researchers to be attuned to how EBM provides moral affordances to practitioners such as Miller, whose patients were well‐poised to become members of biosocial communities, whereas the same tools may fall flat for those such as Melnyk, whose patients' anxieties were limited to the domain of the personal. I did not conduct cross‐field comparisons, but future research in medical sociology ought to map the plurality of moral backgrounds across medical specialities by interviewing practitioners about the moral dynamism of the epistemic tools that they use to manage the uncertainties endemic to their fields. This comparative work would also be aided by scholarship that traces our understanding of contemporary moral backgrounds to those of the past, a project that Hanemaayer (2020) recently began with her genealogy of EBM.

For those in the burgeoning field of the sociology of morality, this study of surgeons reveals that epistemology is an understudied dimension of the moral background. CPGs may be the last place that one expects to find moral dynamism, but, if surgeons are any guide, knowledge objects can serve as codifications of a moral order, affronts to it, and battlegrounds where moral duty is contested. In surgery, epistemic artefacts have this moral potentiality because they not only aspire to truth, but also create moral solidarity in the form of biosociality (Rabinow 1992), reallocate moral responsibility (Heimer and Staffen 1998) to new actors and disrupt moral boundaries (Lamont and Molnár 2002) between professions.

This paper also invites surgeons themselves, other healthcare professionals, policymakers, and anyone who is involved in drafting evidence‐based CPGs to consider how their products may interact with the moral background. These actors have long recognized the tension between the epidemiological basis of EBM and the clinical expertise of physicians (Sackett et al. 1996), but should also be attuned to how CPGs reproduce moral assumptions, become moral tools and shape the moral roles that actors imagine for themselves. The moral background may be intangible, but it becomes visible in eras when axioms like interventionism are overturned, moments when actors must give local meaning to abstract knowledge, and places where clashing moral precepts meet. Today's CPGs will be the foundation of tomorrow's moral background.

## Author Contributions

**Clay Davis:** conceptualization, data curation, formal analysis, funding acquisition, investigation, methodology, writing – original draft preparation, writing – review and editing.

## Conflicts of Interest

The author declares no conflicts of interest.

## Data Availability

The data that support the findings of this study are not available due to privacy or ethical restrictions.
